# Chick Embryo Growth Modeling Using Near-Infrared Sensor and Non-Linear Least Square Fitting of Egg Opacity Values

**DOI:** 10.3390/s20205888

**Published:** 2020-10-17

**Authors:** Alin Khaliduzzaman, Ayuko Kashimori, Tetsuhito Suzuki, Yuichi Ogawa, Naoshi Kondo

**Affiliations:** 1 Laboratory of Bio-Sensing Engineering, Kyoto University, Kyoto 606-8502, Japan; ts@kais.kyoto-u.ac.jp (T.S.); ogawayu@kais.kyoto-u.ac.jp (Y.O.); kondonao@kais.kyoto-u.ac.jp (N.K.); 2 Faculty of Agricultural Engineering and Technology, Sylhet Agricultural University, Sylhet 3100, Bangladesh; 3 JSPS Postdoctoral Research Fellow, Laboratory of Bio-Sensing Engineering, Kyoto University, Kyoto 606-8502, Japan; 4 NABEL Co., Ltd., Kyoto 601-8444, Japan; kasimori@mail.nabel.co.jp

**Keywords:** chick embryo, growth functions, curve fitting, near-infrared sensor, animal welfare

## Abstract

Non-destructive monitoring of chick embryonic growth can provide vital management insights for poultry farmers and other stakeholders. Although non-destructive studies on fertility, hatching time and gender have been conducted recently, there has been no available method for embryonic growth observation, especially during the second half of incubation. Therefore, this work investigated the feasibility of using near-infrared (NIR) sensor-based egg opacity values—the amount of light lost when passing through the egg—for indirectly observing embryo growth during incubation. ROSS 308 eggs were selected based on size, mass and shell color for this experiment. To estimate the embryo size precisely, we fit various mathematical growth functions during incubation, based on the opacity value of incubated eggs. Although all the growth models tested performed similarly in fitting the data, the exponential and power functions had better performances in terms of co-efficient of determination (0.991 and 0.994 respectively) and RMSE to explain embryo growth during incubation. From these results, we conclude that the modeling paradigm adopted provides a simple tool to non-invasively investigate embryo growth. These models could be applied to resolving developmental biology, embryonic pathology, industrial and animal welfare issues in the near future.

## 1. Introduction

Non-destructive monitoring of chicken eggs during incubation can provide vital management insights for avian scientists, poultry farmers and other stakeholders. Although non-destructive studies on fertility [1,2,3], heart beats [4,5] and hatching time [4,6,7] observation have been conducted recently, there is no method available to date to observe the embryo growth during the second half of incubation. Chicken embryos develop within the confines of the eggshell from a fertilized ovum to a functional hatchling chick. During this incubation period, the embryos grow rapidly, increasing many hundredfold in mass [8]. It is important to observe embryo growth for several reasons, in terms of commercial viewpoints, evolutionary ecological importance and animal welfare issues. To date, however, there have been limited in-depth studies of eggs during incubation, in terms of embryo growth. The only study previously carried out on embryo growth used a visible transmission-based spectroscopy [9]. This research was conducted over a very narrow incubation period, days 5 to 10, because of the limitations of the spectroscopic method.

The spectroscopic method is a kind of first generation optical sensor, which has less potential in many biological cases such as the study of hatching eggs in the second half of incubation, as the egg becomes too opaque for visible light to pass through [9]. However, observing embryo growth over a wider range of the incubation period, especially in the second half, is very important. This is because the latter half is the “growth phase”, whereas the first half is referred to as the “formation phase” [10,11]. Embryonic growth estimation is more important after day 10 of incubation, since the developing embryo enters into the growth phase during the second half of incubation. Moreover, exponential growth patterns and differences among embryos are more visible in the last half of incubation. The embryo gains most of its weight in the second half of incubation due to the nature of exponential growth in vertebrates. Thus, our research aims to evaluate the feasibility of using near-infrared (NIR) sensor-based opacity (the amount of light lost when passing through the egg) value to indirectly observe the embryonic growth of the chick. NIR light is used because it has a greater transmission rate than that of visible light in the latter half of incubation [12].

By observing embryonic growth, many issues can be highlighted. For example, slowing embryonic growth prolongs exposure and thereby increases the risk of predation and other mortality factors, and variation in embryo growth rate has important implications for evolutionary fitness [13]. Thus, the embryo growth history could have significant correlations with the post-hatch growth performance of birds. Observation of embryonic growth during incubation is also important from a humane treatment viewpoint, since abnormal, small or slow-growing chicks are normally culled after hatching due to their inferior growth rate and post-hatch performance. Therefore, it is very important to develop a protocol that enables us to non-destructively monitor embryonic growth during incubation. Many growth models are available for birds and mammals: exponential, sigmoid, power and Gompertz functions are popular and frequently used for growth observation in vertebrates [8,13,14]. None of these functions have been used or adopted for non-destructive embryonic growth observation in the past. This is because all these models require the actual embryo growth rate to be fitted with mathematical models. 

In this respect, optical methods have clear advantages in an industrial setting compared with other non-invasive methods. Moreover, the captured optical spectrum contains information about the structure of the biological sample [15]. Because the eggshell and the embryo within have a high absorbance in the UV and visible region respectively, it is thought that near-infrared (NIR) light is likely a better option for studying hatching eggs and quantifying embryo growth, since 600 to 900 nm wavelengths have lower absorbance through chicken hatching eggs [16].

Based on the above requirements, the present research was designed to develop a new technique and define an incubation life history variable, the opacity of hatching eggs, as a means of studying embryonic growth during incubation. It was assumed that light absorbance would be higher for larger embryos. Secondly, as the embryo components become larger, the amount of light absorbance would be increased, hence the photodiode current and, subsequently, the output voltage would decrease. Consequently, the opacity, the ratio of input LED current over average output voltage, was hypothesized to increase, and was used to study embryo growth during incubation in this research.

## 2. Materials and Methods

### 2.1. Materials

This animal experiment was conducted according to the ethical standard of Kyoto University (Approval number: 28–59). Eggs from mother birds of the same age (54 weeks old parent stock) and the same broiler strain (ROSS 308) were used in this experiment. Eggs were supplied by Yamamoto Co. Ltd. at Kyoto, Japan. Before incubation, eggs were graded based on major axis (59.5 ±3.0 mm), minor axis (46 ±1.0 mm), weight (68 ±5.0 g) and machine-vision-based eggshell color (*r* = 0.375 ±0.015) to minimize the egg size and eggshell color variations among egg samples. Temperature at 18.0 (±0.5) °C and relative humidity of 80% (±5%) were maintained during the pre-incubation storage of five days [5]. 

### 2.2. Experimental Protocols

The sorted eggs were set in an incubator with automatic turning (90° per hour) operation (SSH-02, Showa Furanki, Japan) at 37.8 °C and 55% RH. Before being put into an incubator, eggs were preheated for 16 h to minimize the thermal shock on embryos. The eggs were transferred to hatcher trays after day 18 at 37.8 °C and 60% RH. The input LED current (*I*_LED_) and average output voltage (*V*_avg_) were recorded once daily for all egg samples using a near-infrared sensor during incubation (days 6–16). The measurement was conducted outside of the incubator, and eggs were immediately returned to the incubator in order to ensure minimal disturbances by the outside environment. 

### 2.3. Near-Infrared Sensor

**Figure 1:**
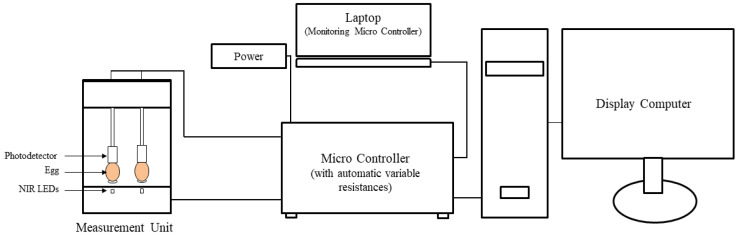
Schematic diagram of near-infrared sensor and signal acquisition system of incubated eggs. The input LEDs light intensity (0.73–96.15 mA) was controlled to keep the output signal within a certain range (3–9.7 V) by 16 variable series resistances in the microcontroller.

A near-infrared sensor which consisted of six LEDs that emit light at peak wavelength 870 nm, as well as a Si photodetector, was used for this experiment (Figure 1). The light received by the photodiode was converted into current and the signal was amplified into a voltage signal using an amplifier. The signal sampling rate per second was 33.3, while the duration of the LED emission cycle was 30 ms. The *V*_avg_ was calculated from the output voltage (*V*_out_) using a 9 s signal for each egg.

### 2.4. Calculation of Egg Opacity Value

Opacity is defined as the ratio of input LED current over average output voltage. The opacity value of an egg increases with the development or changes of embryonic components, such as embryo, allantois, yolk sac and other structures. The higher the opacity value, the higher the input current (i.e., higher input light intensity) is necessary to get the same output voltage. As opacity refers to the amount of light loss during transmission through the egg sample, it is something close to absorbance. In spectroscopy, absorbance (*A* α *I*_o_/*I* and transmittance, *T* α *I*_out_/*I*_in_, where *I*_out_ is transmitted light intensity and *I*_in_ is incident light intensity) is measured based on light intensity. In the case of the electrical circuit/device (e.g., NIR sensor in our experiment), the input and output current are slightly different from the light intensity and thus need to be defined by either current (*I*_LED_) or voltage (*V*). Hence, opacity may be defined as the ratio of input current (*I*_LED_) and output current. However, input current is not proportional to output current, but rather is proportional to output voltage (*V*_out_).

(1) $$\mathrm{Opacity}=\frac{\mathrm{LED}\;\mathrm{input}\;\mathrm{current},\;\mathrm{mA}}{\mathrm{Average}\;\mathrm{output}\;\mathrm{voltage},\;V}=\frac{I_{\mathrm{LED}}}{V_{\mathrm{avg}}}$$

where *V*_avg_ is the average of output voltage, *V*_out_

(2) $$\mathrm{Average}\;\mathrm{output}\;\mathrm{voltage},\;V_{\mathrm{avg}}=\frac{1}{290}\overset{300}{\underset{n=11}{\sum }}V_{\mathrm{out}}$$

where *n* is the sample count of 33.3 Hz signal. The first 10 sampling points were used for adjusting resistance and input current for the LED to reach an output voltage between 3 and 10 V.

The weight of neonatal chicks after hatching was measured as supporting data using a digital balance. The chick conversion ratio was calculated based on chick weight divided by the initial egg weight.

### 2.5. Data Analysis and Curve Fittings

The growth process of avian embryo can often be approximated by decaying exponential functions of time, such as the logistic, Gompertz and power functions [8,13,14]. Therefore, mathematical growth models, such as exponential, sigmoid, power and Gompertz functions, were used for fitting the hatching egg opacity from day 6 to 16 using non-linear least square curve fitting. Growth was estimated as a function of opacity and incubation time (*t*). MATLAB software (2020a) was used for all data analysis and non-linear least square curve fittings. The MATLAB algorithm keyword “[x, resnorm] = lsqnonlin (fun, x0)” and Levenberg–Marquardt algorithm were used for this purpose. It required an initial point x0 and a function fun to compute the vector-valued function. 

After grading eggs based on size (minor and major axis), weight and shell color, the eggs were divided into two groups: Large (L), and Extra Large (XL), based on the following Equations (3) and (4) (Table 1).

(3) $$W_{L}<W_{\min}+\frac{W_{\max}-W_{\min}}{2}$$

(4) $$W_{\mathrm{XL}}>W_{\min}+\frac{W_{\max}-W_{\min}}{2}$$

where *W*_max_ was 72.48 g and *W*_min_ was 61.72 g. The same principle was applied to categorize other groups, such as egg dimension and chick weight. 

After curve fitting with various mathematical growth models, the calculated opacity value was correlated with embryo weight reported by Byerly [17]. The evaluation of the various growth models was based on the coefficient of determination and RMSE. 

## 3. Results

### 3.1. Physical Meaning of Opacity Curve

**Figure 2:**
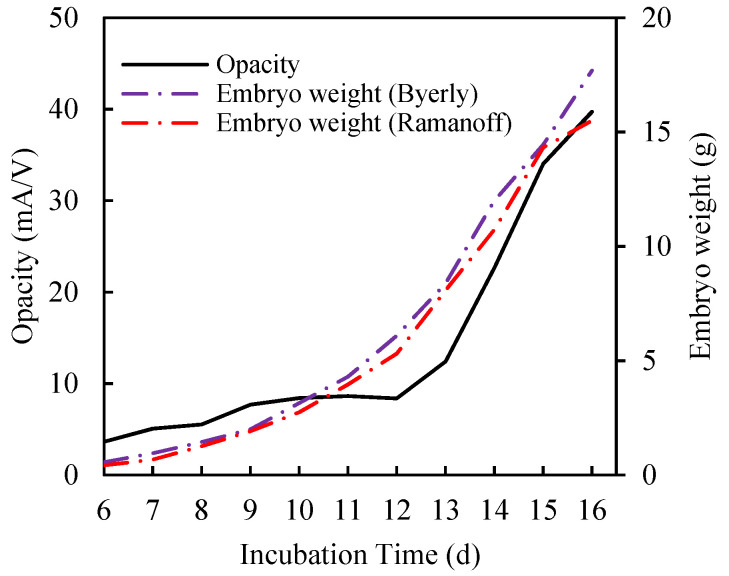
Changes in opacity and embryo weight during incubation. There were almost similar trends in near-infrared (NIR) opacity value and embryo weight change during incubation.

Simply put, opacity is a measure of incubated hatching egg absorbance. However, the unit is different from absorbance. Absorbance is widely used in optical sensors, but opacity is defined here by an electrical/optoelectrical sensor. Common spectroscopic techniques cannot be applied to incubated hatching eggs after day 10 of incubation because of high opaqueness. The concept of using opacity is as follows: as the embryonic components within the egg become larger during incubation, the amount of transmitted light is reduced. Consequently the opacity, that is, the ratio of input LED current over average output voltage, increases [18]. The opacity of incubated hatching eggs during incubation increased more or less exponentially, peaking at day 15–16 before slowing (Figure 2). During the first half of incubation (until day 10), the rate of increase was relatively slow compared to that in the second half, since the embryos are relatively small and immature during this stage, referred to as the formation phase. The opacity of an incubated hatching egg is largely influenced by embryo size, followed by allantois, yolk and yolk sac. During this formation phase, the growth rate of the embryo is much lower than that during the last half of incubation, referred to as the growth phase. Subsequent rapid growth of the embryo from day 12 to 16 of incubation is thought to be responsible for the rapid changes seen in opacity during this period.

On the other hand, embryo weight gain follows an exponential or sigmoid curve, where the rate of growth is much slower during the first half of incubation, followed by rapid growth in the second half of incubation (Figure 2). Finally, at the very end of the incubation period, the rate of growth begins to slow down. This is a common growth phenomenon in vertebrates. The embryo weights separately observed by Byerly and Romanoff for precocial birds are very similar [17,19].

### 3.2. Growth Dependency of Opacity Curve

#### 3.2.1. Egg Size

**Figure 3:**
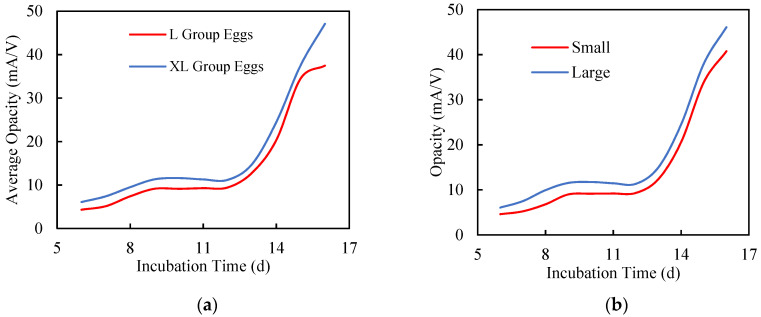
Patterns of opacity curves for various groups of eggs during incubation (days 6–16). Longer major axis eggs had higher opacity due to a longer optical path. (**a**) Growth pattern of embryos from small and large eggs. (**b**) Growth pattern of small and large major axis eggs.

Transmission based on the near-infrared signal was used to define opacity, which is influenced by egg size (mass and major axis of egg) as shown in Figure 3. Larger eggs generally have a higher opacity, as these eggs contain more internal matter and produce larger embryos. As the embryonic components became larger during incubation, the amount of transmitted light was lower, hence the photodiode current, and subsequently the output voltage, decreased. Consequently, opacity increased. Reduced transmission (higher opacity) results from dispersion of the NIR light by hatching eggs, which depends on variations in the growth of the embryo, the yolk sac and the allantois, as well as their relative orientation. The opacity is also influenced by the major axis of the egg (Figure 3b). Eggs with a greater major axis length had higher opacity than those with shorter major axis length due to the longer optical path (when vertical optical configuration was used).

#### 3.2.2. Chick Size

**Figure 4:**
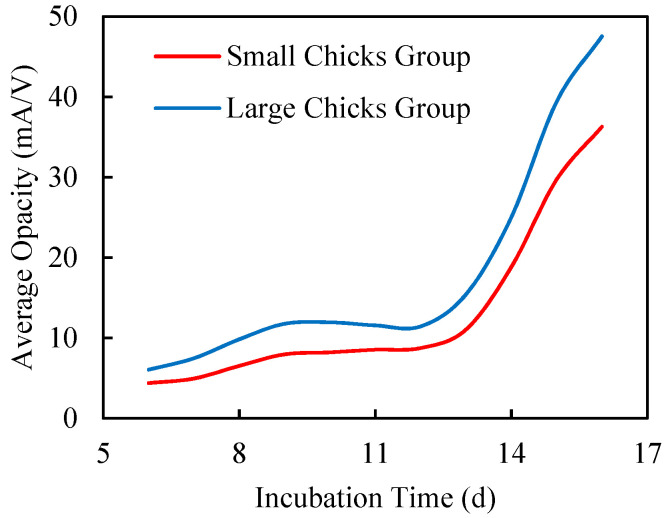
Growth pattern of small and large size chick groups during incubation (days 6 to 16).

Opacity is mostly influenced by embryonic components during incubation, of which embryo size is the predominant factor. This is witnessed by the fact that the small chick group had a lower average opacity value than the large chick group over the incubation period (Figure 4). The larger the embryo, the higher the opacity due to higher absorbance. Although eggs were graded based on size, weight and shell color, there were variations in chick embryos. In some cases, relatively large eggs produce relatively small chicks because of lower conversion ratios (i.e. chick weight/egg weight) of internal egg resources, and perhaps as a result of hypoxia, beside genetic factors. Hypoxia during incubation decreases growth rate and body size in a variety of species [20,21]. Conversely, embryo growth can be accelerated by maintaining an appropriate oxygen concentration during incubation, typically 60% [22]. Therefore, non-destructive embryonic growth observations during incubation can also provide valuable information about impending hypoxia and the need for intervention for the recovery of embryonic growth. 

### 3.3. Curve Fitting and Mathematical Growth Models

When performing curve fitting, several important points need to be considered. Firstly, the opacity value reflects growth information from all embryonic components (embryo size, yolk sac, allantois). Therefore, fitting a mathematical function may eliminate the influence of yolk sac and allantois on the opacity curve. This is because embryo growth generally follows an exponential, sigmoid or power function. Secondly, embryo growth needs to be monitored non-invasively if it is to be more precise. However, a clear deviation was seen between opacity points and fitted curve, especially from day 7 to day 12 of incubation, due to the influence of allantois. 

**Figure 5:**
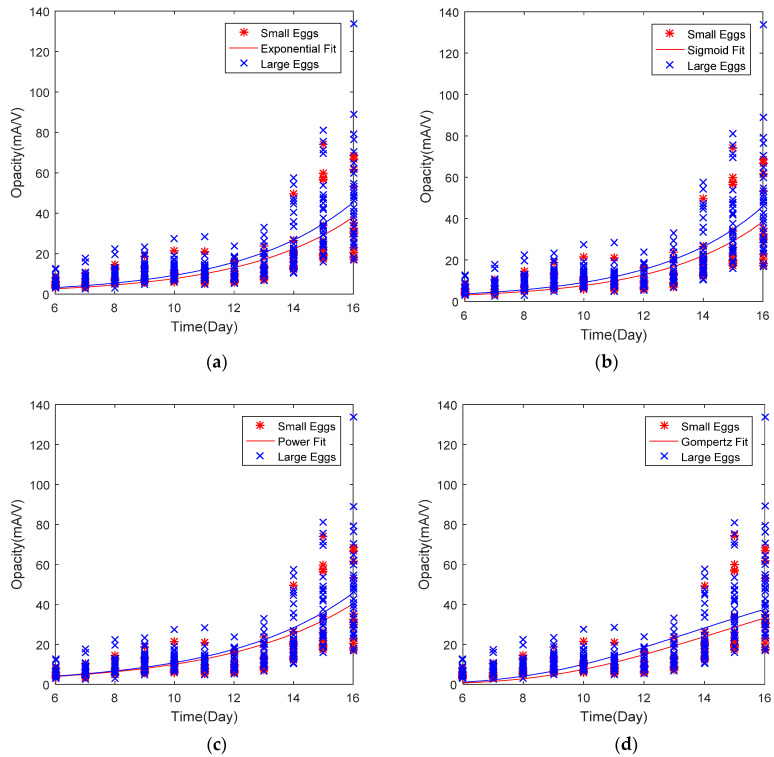
Non-linear least square fitting of opacity curves using various mathematical growth models. (**a**) Exponential fit of opacity data of egg samples, (**b**) Sigmoid fit of opacity data of egg samples, (**c**) Power function fit of opacity values of eggs, (**d**) Gompertz function fitting of incubated egg opacity values for both groups of embryos. The fitting may eliminate the influence of allantois and yolk sac growth during the first half of incubation (formation period).

**Figure 6:**
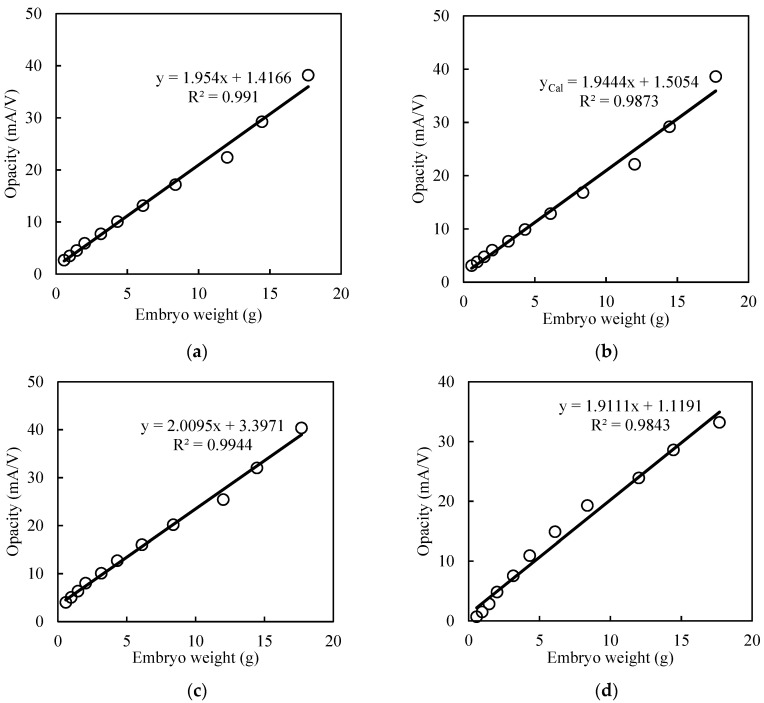
Correlation between calculated opacity by various mathematical models and embryo weight (small eggs group). (**a**) exponential fit, (**b**) sigmoid fit, (**c**) power fit and (**d**) Gompertz fit. The embryo weight used is from the experiments of Byerly [17].

The smaller size egg group had a lower average value of opacity over the incubation period than the larger egg group in all mathematical models (Figure 5). This makes sense, as chick embryos rapidly gain body weight in the second half of incubation and thus the differences gradually increase over time. After curve fitting with various mathematical growth models, the calculated opacity as a function of time (*y* = *f*(*t*)) was correlated with the embryo weight reported by Byerly [17]. The calculated values of opacity for various models showed a high correlation with embryo weight (Figure 6).

Therefore, the embryo weights could be calculated from the calculated opacity value using exponential, sigmoid, power and Gompertz growth models (Equations (5)–(8)).

(5) $$x=\frac{y_{\mathrm{exponential}}-1.4166}{1.954}$$

(6) $$x=\frac{y_{\mathrm{sigmoid}}-1.5054}{1.9444}$$

(7) $$x=\frac{y_{\mathrm{power}}-3.3971}{2.0095}$$

(8) $$x=\frac{y_{\mathrm{Gompertz}}-1.1191}{1.9111}$$

where *x* is embryo weight, and *y* is calculated opacity obtained by fitting various mathematical models.

The performances of the various equations are shown in Table 2. The exponential and power function models had higher performance in explaining embryo growth in terms of the coefficient of determination, R^2^ = 0.991 and 0.994, respectively. 

The above equations came after optimization by minimizing the error between actual opacity and calculated opacity at certain days of incubation (Equations (5)–(8)). Coefficients (parameters) *a* and *b* are calculated based on minimization of the error with multiple iterations. The Gompertz function is defined by M $=Ae^{-be^{-ct}}$, where M is embryo mass, *A* is the upper asymptote and *a* and *b* are the growth rates. The Gompertz function is a kind of sigmoid function. It is a type of time series mathematical model, where growth is slowest at the start and end of a time period (Figure 5d). The upper asymptote was 70 mA/V, the opacity value of the eggs.

All mathematical models were almost equally acceptable in terms of performance parameters such as RMSE and coefficient of determination (Table 2). The lowest RMSE was for the power function, since the trend in the data was more similar to that of the power function curve. The Extra Large (XL) eggs group had higher variations in opacity and a greater number of samples. Calculated opacity values fitted with exponential and power functions performed better in terms of the coefficient of determination with embryo weight (Figure 6). Although the Gompertz function had a lower performance in fitting opacity data, it provided a clearer idea of the embryo weight as this value was very close to embryo weight values (Equation (8)).

Although embryo size is highly correlated with egg size, if graded eggs are used, egg size might not always provide precise information about embryo weight or size during incubation. This is because two eggs of the same size can produce different sizes or weights of hatchling chicks. This variation may come from different conversion ratio, hypoxia, gender, or resources allocated inside the eggs (e.g., ratio of egg yolk and albumen). Thus, the technique developed for embryonic growth observation in this research may solve this issue.

One limitation of this study is that it estimated growth only until day 16 of incubation. This technique or wavelength may not be suitable for studying growth in the last few days of incubation. Researchers may need to look for another approach to study this range in the future. During this very late stage of incubation, the internal structure of the egg is very different—it is relatively dry, feathers are fully developed and shell thickness is becoming much thinner, and thus much higher transmission results in lower opacity values. Another concern is regarding embryo exposure to high voltage or current. Near-infrared light is relatively safe for living organisms, especially if the exposure time is short. No adverse effects were observed regarding hatchability, since the hatch rate was more than 90%, including infertile eggs. Generally, about 5–7% of incubated eggs are found to be infertile. However, it is not recommended to apply a higher input current or light intensity for longer than 10 s for the inspection of hatching eggs, especially before day 10, due to the tiny embryo size and higher sensitivity to external stresses.

## 4. Conclusions

From a commercial viewpoint, the estimation of embryo growth before hatching is a crucial challenge for poultry farmers and other stakeholders worldwide. Monitoring embryonic growth during incubation is also important from a humane treatment viewpoint, since abnormal, small or slow-growing chicks are normally culled after hatching due to their inferior growth rate and inferior post-hatch performance. This research evaluated the use of mathematical growth models based on the opacity of incubated hatching eggs to non-destructively estimate chick embryo weight during incubation. Although all models performed well, the power function model had the highest performance in explaining embryo weight in terms of the coefficient of determination, R^2^ = 0.994. This research is an important step towards the study of avian embryos in the field of physiology, developmental biology, pathology, animal ecology and precision poultry production systems, since it enables non-invasive monitoring of the growth of individual embryos. This novel technique can also be used to advance avian and reptile research.

## Figures and Tables

**Table 1:** Criteria for making various groups of egg and chick size.

Groups	Egg Weight, g	Chick Weight, g
L (Large) Eggs	<67.1 g (*n* = 13)	
XL (Extra Large) Eggs	>67.1 g (*n* = 33)	
Small Chicks		<50.0 g (*n* = 14)
Large Chicks		>50.0 g (*n* = 32)

*n* is sample size.

**Table 2:** Equation, parameters and performances of various growth models with respect to opacity and embryo weight.

Model Name	Eggs Group	Equation	RMSE (Opacity)	R^2^ (Fitting Curve)	R^2^ (Cal. Opacity vs. Embryo Wt.)
Exponential	*L-Size*	$y=0.5357e^{0.2667t}$	14.85	0.558	0.991
	*XL-Size*	$y=0.6602e^{0.2645t}$	18.14	0.568	0.991
Sigmoid	*L-Size*	$y=\frac{1}{1+0.3718e^{0.2885t}}$	14.82	0.561	0.987
	*XL-Size*	$y=\frac{1}{1+0.4777e^{0.284t}}$	18.15	0.573	0.988
Power	*L-Size*	$y=1.26^{t}$	12.51	0.529	0.994
	*XL-Size*	$y=1.27^{t}$	16.78	0.561	0.994
Gompertz	*L-Size*	$y=70e^{-13.8e^{-0.1823t}}$	15.42	0.504	0.984
	*XL-Size*	$y=70e^{-12.95e^{-0.1898t}}$	18.66	0.487	0.972

Opacity, *y* = *f*(*t*) where *t* is incubation time (day).
